# Molecular Decoding of Phytohormone Crosstalk: JA-Mediated Key Regulatory Nodes and Signal Integration

**DOI:** 10.3390/plants14172647

**Published:** 2025-08-26

**Authors:** Hui Gan, Shiying Wang, Zisong Yang, Pengda Ma

**Affiliations:** 1 College of Resources and Environment, ABA Teachers College, Wenchuan 623002, China; wsy891023@outlook.com; 2 College of Life Sciences, Northwest Agriculture and Forestry University, Yangling 712100, China; ganhui@nwsuaf.edu.cn

**Keywords:** jasmonates, hormone crosstalk, molecular mechanism, plant growth, plant stress responses, JA signaling

## Abstract

Jasmonates (JAs) are crucial phytohormones governing plant growth and defense against stresses. This review synthesizes the intricate molecular mechanisms underlying JA crosstalk with key hormones: auxin (AU), gibberellin (GA), abscisic acid (ABA), ethylene (ET), brassinosteroids (BRs), strigolactones (SLs), and salicylic acid (SA). We focus on interactions during development and stress adaptation, highlighting how these range from synergistic (e.g., JA-ABA/ET in defense, JA-AU in root growth) to antagonistic (e.g., JA-SA in pathogen response, JA-GA/BRs in growth processes). Central to this crosstalk are key regulatory nodes like the MYC2 transcription factor and JAZ repressor proteins, which integrate signals through transcription factor networks, targeted protein degradation, and post-translational modifications. By elucidating these molecular pathways, our review establishes a framework for understanding the complex regulatory logic of hormone interactions. Furthermore, it offers insights for the strategic engineering of hormone signaling (e.g., modulating JAZ stability or MYC2 activity) to enhance crop resilience to environmental challenges.

## 1. Introduction

Jasmonates (JAs) are essential lipid-derived phytohormones that orchestrate plant development and stress adaptation. Crucially, JAs function not in isolation but through dynamic crosstalk with other hormonal pathways (e.g., auxin, gibberellins, ABA, ethylene, salicylic acid) to fine-tune responses to environmental fluctuations and developmental cues. This crosstalk enables plants to prioritize resource allocation between growth and defense—a central theme in plant fitness [1].

To date, three pathways for JA biosynthesis have been revealed: (1) the octadecanoid pathway starting from α-linolenic acid (α-LeA, 18:3); (2) the hexadecanoid pathway starting from hexadecatrienoic acid (16:3); and (3) the OPDA reductase 3 (OPR3)-independent pathway. In *Arabidopsis thaliana*, the first two pathways are predominantly localized in mesophyll cells, where polyunsaturated fatty acids (e.g., α-LeA and 16:3) are released from chloroplast membranes (in mature leaves) or pro-plastid membranes (in developing tissues) and then converted to OPDA and dn-OPDA in plastids. These intermediates are subsequently transported to peroxisomes of the same mesophyll cells for β-oxidation, ultimately yielding JAs [2,3,4].Crucially, JA biosynthesis occurs primarily in photosynthetic tissues, with mesophyll cells serving as the main sites due to their high chloroplast density and peroxisomal abundance. This process is strongly induced by environmental stresses such as mechanical wounding, herbivory, and pathogen attack, as well as during specific developmental stages (e.g., flower maturation and root growth) [5,6,7]. JAs undergo conjugation and modification within the cytoplasm; then, bioactive jasmonoyl-isoleucine (JA-Ile) is produced by the enzymes Jasmonate Resistant 1 (JAR1)/GH3.11 and GH3.10. Methyl jasmonate (MeJA) and 12-hydroxyjasmonic acid (12-OH-JA) are further derivatives [8].

Jasmonoyl-isoleucine (JA-Ile) can be transported into the nucleus by the ABCG transporter Jasmonate Transporter 1 (JAT1). Upon perception of JA-Ile by the SCF complex (SCF^COI1^)—an E3 ubiquitin ligase complex comprising COI1, ASK1/ASK2, and *AtCUL1*—this complex mediates the ubiquitination of JAZ proteins and triggers their degradation via the 26S proteasome. This process triggers the JAZ-mediated repression of downstream transcription factors, thereby activating the jasmonate signaling cascade. Consequently, plants gain the capacity to regulate growth and development or respond to biotic/abiotic stresses. This mechanism constitutes the canonical JA signal transduction pathway, wherein JAZ proteins function as core transcriptional repressors [9,10]. The F-box protein Coronatine Insensitive 1 (COI1), bHLH transcription factors, MYB transcription factors, transcription factors from other hormone signaling pathways (e.g., EIN3, EIL, GAI, RGA, and RGL1), and co-repressors (e.g., NINJA, TPL, HDA6, and HDA19) all can interact with JAZ [11,12].

Hormones in plants are essential in controlling abiotic stress responses, flowering, senescence, dormancy, and seed germination. Research has demonstrated that JA interacts with AU, GA, ABA, ET, BRs, SLs, and SA to control plant development and stress responses. Plants are better able to adapt to their surroundings thanks to these interactions [13]. The JA response is primarily mediated by the JA signaling repressor JAZ protein family, which regulates JA-controlled signaling pathways. JAZ proteins regulate activities like seed germination, the development of root hair and floral organs, trichome elongation, and stress responses by directly or indirectly suppressing the transcriptional activity of target genes or proteins [14,15].

This review focuses on synthesizing established molecular mechanisms of JA crosstalk with other hormones, highlighting key nodes of crosstalk and exploring the regulatory mechanisms at different molecular levels. While broader questions persist in the field, key unresolved challenges encompass the poorly resolved spatiotemporal regulation of dynamic hormone gradients and tissue-specific signaling hubs (e.g., root versus leaf JAZ isoforms) [16], the need for the deeper exploration of epigenetic control via histone modifications regulating JA-responsive genes (e.g., MED25-MYC2) [17], and the underexplored mechanisms in non-model plants despite their agronomic relevance [18]. These critical frontiers extend beyond the scope of our mechanistic synthesis but will be discussed as essential future research directions in the Section 3.

## 2. Molecular Mechanisms of Jasmonate Interactions with Other Hormones

### 2.1. JA and ABA Crosstalk: JAZ-MYC2

Previous studies have shown that the plant hormone ABA mediates developmental processes and tolerance to abiotic stresses, particularly drought and salinity [19,20]. The ABA signaling pathway is similar to the “de-repression model” of JA signaling. When the ABA receptors belonging to the Pyrabactin Resistance/Regulatory Component of Abscisic Acid Receptor (PYL/RCAR) family detect ABA signals, they establish stable complexes with type 2C protein phosphatases (PP2Cs) [21]. This interaction results in the release of SNF1-related kinases 2 (SnRK2s), which are normally inhibited by PP2Cs, activating downstream transcription factors such as Abscisic Acid Insensitive 5 (ABI5) and ABA Response Element Binding Protein/ABRE Binding Factor (AREB/ABF) [4].

**Figure 1:**
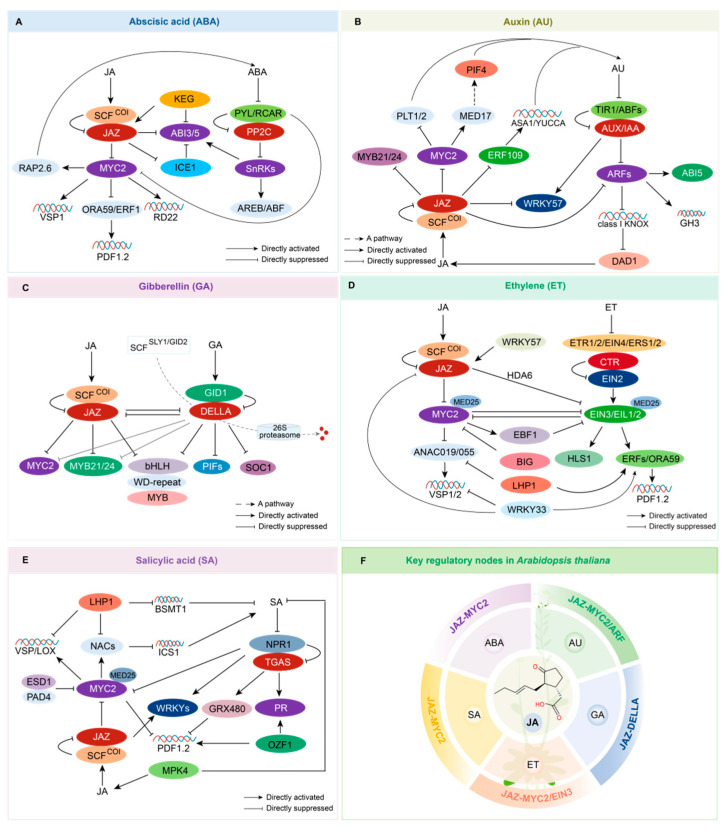
Molecular mechanisms of JA and other hormones’ crosstalk in *Arabidopsis thaliana*. (**A**) Molecular mechanisms of jasmonic acid and ABA crosstalk. JAZ proteins and the MYC2 transcription factor are key nodes in crosstalk, regulating plant growth and development, defense processes, and responses to abiotic and biotic stresses. *RD22*: a drought-responsive gene; *PDF1.2*: a pathogen defense gene; *VSP1*: a herbivory defense gene; RAP2.6: a transcription factor involved in the wound healing process. (**B**) Molecular mechanisms of jasmonic acid and auxin crosstalk. JAZ proteins, MYC2, and ARF transcription factors are key nodes in crosstalk, regulating plant growth and development, particularly root development, as well as defense responses. PIF4: involved in light signaling and growth regulation; *ASA1/YUCCA*: genes related to auxin biosynthesis; *GH3*: a gene regulating jasmonate homeostasis; *DAD1*: a gene involved in jasmonate biosynthesis. (**C**) Molecular mechanisms of jasmonic acid and GA crosstalk. JAZ proteins and DELLA proteins are key nodes in crosstalk, regulating plant growth and development, particularly floral development, as well as defense responses. PIFs (phytochrome interacting factors): transcription factors that regulate hypocotyl elongation in plants; SOC1 (Suppressor of Overexpression of Constans 1): a regulator of flowering in plants; *DAD1/LOX* (lipoxygenase): genes involved in the biosynthesis of JA. (**D**) Molecular mechanisms of jasmonic acid and ethylene crosstalk. JAZ proteins, MYC2, and EIN3 transcription factors are central nodes in crosstalk, regulating plant growth and development, as well as defense responses. HLS1: a transcription factor involved in hook development; MED25: a key subunit involved in plant hormone signaling and the regulation of gene expression; BIG: a protein involved in hormone responses; ANAC019/055: NAC transcription factors that participate in plant stress and defense responses. (**E**) Molecular mechanisms of jasmonic acid and SA crosstalk. The MYC2 transcription factor and the NPR1 receptor are key nodes, primarily involved in regulating plant defense responses. *ICS1*: a gene related to the biosynthesis of SA; *BSMT1*: a gene encoding a key enzyme in SA metabolism; LHP1: a key member of the Polycomb histone complex, involved in the repression of gene expression; OZF1: a positive regulator in the SA signaling pathway. (**F**) Key regulatory nodes in *Arabidopsis*.

In the intricate signaling networks governing plant responses to biotic and abiotic stresses, including drought, the interplay between ABA and JA is predominantly mediated by the transcription factor MYC2 and JAZ proteins (Figure 1A). Under drought stress, elevated ABA concentrations trigger the degradation of specific JAZ proteins (notably JAZ3 and JAZ12), alleviating the suppressive effect on MYC2 and enabling its positive regulation of the *RD22* gene expression in root cells (vegetative tissues) [22,23]. In leaf mesophyll cells, ABA and JA act on MYC2 to synergistically repress the pathogen defense pathway mediated by ERF1/ORA59-PDF1.2, but they cooperatively promote the insect defense pathway mediated by MYC2-*VSP1* [24]. PYL6 specifically interacts with MYC2 in the quiescent center (QC) and surrounding stem cell niche cells of root apical meristems. This interaction attenuates JA-mediated stem cell activation under abiotic stress, thereby optimizing resource allocation between growth and stress adaptation. Specifically, the PYL6-MYC2 complex inhibits MYC2’s transcriptional activation of *PLT1/2* and ERF109, which are essential for root regeneration and defense responses [25]. The transcription factors MYC2/3 boost lignin deposition during plant wound recovery by activating RAP2.6 and promoting ABA biosynthesis [26].

Additionally, SnRK kinases are a key component in ABA signaling, and the JA-ABA module dynamically regulates hormone biosynthesis to achieve hormonal crosstalk. The ZmSnRK2.2-ZmHsf28-ZmJAZ14/17 module dynamically regulates oxygen species accumulation in response to drought stress in leaf mesophyll cells [27]. ABA inhibits seed germination by stimulating the ABA regulators ABI3 and ABI5, which is based on the breakdown of JAZ proteins and the stimulation of SnRK [14]. The MYC2-ABI5 complex upregulates the expression of β-GLUCOSIDASE18 (BGLU18), thereby promoting increased abscisic acid (ABA) biosynthesis, which enhances root regeneration under stress conditions [28]. Researchers identified the “SAPK10-bZIP72-AOC” pathway, revealing that SnRK activates the bZIP72 transcription factor to upregulate the *AOC* gene, thereby promoting JA biosynthesis [29]. However, wheat research demonstrated that JA lowers ABA levels and adversely affects seed dormancy by controlling the expression levels of particular ABA biosynthesis (*NCED*) and catabolism (*cyp707a*) genes [21]. JA production is encouraged by the ABA-responsive transcription factor AchABF1-1, which also favorably controls the buildup of suberin polyphenols during kiwifruit wound healing [30].

The integration of the JA-ABA hormone cross-regulatory network includes other regulatory variables. As an inhibitor of the ABA-dependent signaling pathway, ICE1 can directly affect seed germination and cold tolerance by interacting with JAZ and the ABI transcription factor [31,32]. Scientists have experimentally demonstrated that, in apples, the ABA-insensitive protein 4 (MdABI4) interacts with MdJAZ, weakening the interaction between MdABI4 and the cold stress regulator MdICE1 to regulate cold tolerance negatively [33,34]. KEG is another crosstalk point in JA and ABA signaling. The E3 ubiquitin ligase Keep on Going (KEG) protein diminishes the suppressive influence of ABA on JAZ12, thereby preserving the stability of JAZ12; moreover, KEG serves as an auxiliary regulator for the bZIP-type transcription factors ABI5, ABF1, and ABF3. It directly interacts with ABI5 to attenuate ABA signaling pathways [34,35,36]. Another JA/ABA dual-responsive transcription factor, AaTCP, was found to interact with the transcription factor AaORA to favorably control the production of artemisinin in *Artemisia annua*, as was the hormone crosstalk connection point AaGSW1 [37]. Recent studies in poplar stated that the AP2/ERF transcription factor PtoERF15 can positively regulate PtoMYC2b to respond to drought stress, although its crosstalk with ABA/JA has not been confirmed [38].

### 2.2. JA and Auxin (AU) Crosstalk: JAZ, MYC2, and ARF

Plant growth and development, as well as the spatiotemporal coordination of signal transduction across tissues and organs, rely predominantly on endogenous auxin biosynthesis mediated by cell type-specific pathways. In *Arabidopsis*, distinct auxin sources—including 15 YUCCA (YUC) flavin monooxygenases and TAA1/TAR aminotransferases—generate local auxin maxima in specific cell types (e.g., root QC cells, shoot apical meristem stem cells) to regulate developmental plasticity in response to environmental cues [39]. There are four categories of auxin receptors known to exist: auxin binding protein 1 (ABP1) and its partner transmembrane kinase (Transmembrane Kinase TMK1-TMK4); Transport Inhibitor Response 1 (TIR1) and its homologs Auxin Signaling F-box proteins (AFB1-AFB5); S-phase kinase-associated protein 2a (SKP2a); and auxin response factor 3 (ARF3, also known as ETTIN or ETT) [40]. Auxin triggers rapid responses through dual perception mechanisms: cell surface-based TMK1 phosphorylation cascades for immediate physiological adjustments (e.g., apical hook formation) and nuclear TIR1/AFB-Aux/IAA degradation for transcriptional reprogramming [41]. These cell type-resolved auxin dynamics enable plants to prioritize organ-specific adaptations, such as root stem cell quiescence under drought (via TAA1-derived auxin) or shoot regeneration upon wounding (via YUC1/4-mediated synthesis) [41,42].

The transcription factors JAZ, MYC2, and ARF serve as the central nodes of the plant JA-AU hormone signaling network (Figure 1B). These variables dynamically control hormone homeostasis in order to coordinate organ growth and environmental reactions. During root development, JA stimulates the MYC2 transcription factor, which interacts with PLT1/2 transcription factors to limit their function. PLT transcription factors indirectly modulate the abundance and polar localization of PIN proteins by sustaining the auxin gradient at the root apical meristem. The gene *PIN2*, which codes for an auxin efflux protein, is expressed less frequently as a result of this effect, changing the distribution of auxin and preventing the formation of main roots [43]. Additionally, the JA signaling system controls AU homeostasis through *YUCCA8/9* expression modulation. The ERF109 transcription factor promotes lateral root elongation during JA induction by upregulating the levels of auxin biosynthesis-related genes *ASA1* and *YUCCA2* (*YUC2*) [44,45]. ARF6/8/17 act on *GH3* genes to regulate JA homeostasis, reducing its activity and stimulating adventitious root formation [46]. During seed germination, ARF10/16 interact with JAZ/ABI5 to integrate the AU-JA-ABA signaling pathways, thereby enhancing ABA-induced delays in seed germination [47]. In floral organ development, ARF6/8 regulate JA signaling through dual mechanisms: (1) inhibiting KNOX I-class genes to promote DAD1-mediated JA biosynthesis and regulate flower development and (2) synergistically activating MYB21/24 with JA to control flower maturation [48]. In apple callus tissue, MdARF8 was discovered to be an inhibitor of JA-induced leaf degeneration and root elongation inhibition [49].

Notably, the JA-AU interaction network forms a senescence-regulatory hub through WRKY57: JAZ4/8 compete with IAA29 for binding to WRKY57, regulating the senescence process through SEN4 and SAG12 [50]. The WRKY57-WRKY33-JAZ1/5 interaction module is also involved in plant disease resistance [51]. JA and AU frequently collaborate in plant defense processes. For example, in *Prunus salicina* infected with black knot (BK), AU stimulated JA accumulation. Similar synergistic effects have been observed in citrus petals infected with anthracnose and tomato virus [52,53,54].

Research on thermomorphogenesis has revealed a non-canonical interaction mode between JA and AU: the MED17 subunit promotes the accumulation of phytochrome-interacting factor 4 (PIF4) in an MYC2-independent JA pathway, thereby upregulating AU to control thermoresponses [55]. This finding expands the functional dimension of JA-AU interactions in environmental adaptation. In summary, the JAZ-MYC2 and WRKY core nodes integrate hormone signals through multi-level regulatory networks to achieve the precise coordination of developmental plasticity and environmental adaptability.

### 2.3. JA and GA Crosstalk: JAZ-DELLA

GA is essential for plant growth and defensive systems: root, stem, leaf, trichome, and flower development. The GA signal network is predominantly mediated by the growth regulator DELLA proteins [56]. When GA is available, the GA receptor GID1 detects and binds to active GA, producing a GA-GID1-DELLA complex including DELLA proteins; this complex then connects with the E3 ubiquitin ligase (SCF^SLY1/GID2^) complex, which ubiquitinates and degrades DELLA proteins via the 26S proteasome pathway; it then reverses the inhibition of downstream transcription factors, enabling plant growth and development [57].

The crosstalk between JAZ and DELLA proteins is the main mediator of the antagonistic and synergistic interactions between the JA and GA signaling networks (Figure 1C). DELLAs competitively bind to JAZ proteins with MYC2, alleviating the inhibition of downstream MYC2 targets [58]. JA can also stabilize DELLAs (e.g., Repressor of GA [RGA]) to regulate downstream genes, such as *PIFs*, which are controlled by the COI1-JAZ-DELLA-PIF signaling module to further regulate hypocotyl elongation [4,59]. A study in rice demonstrated that OsJAZ9 interacts with SLR1 to mediate the antagonistic interaction between JA and GA signals [60]. In cotton, JAZ proteins act downstream of GbWRKY1 to regulate the transcriptional activity of SOC1 in response to pathogen infection [61]. JAZ proteins interact with DELLA proteins and MYC2, thereby enabling JA and GA to synergistically promote the expression of terpene synthase genes (*TPS21* and *TPS11*) [62]. Additionally, MYC2 can induce GA metabolism, while GA negatively regulates the transcriptional accumulation of MYC2. When rice is attacked by brown planthoppers (BPH), OsMYC2 upregulates the expression of GA catabolic genes *GA2ox3/5/7*, thereby promoting GA catabolism. In tomatoes, GA can antagonize the JA signaling network to regulate responses to potassium deficiency. DELLA proteins negatively regulate MYC2 activity, thereby downregulating the expression of steroidal glycoalkaloid (SAG) biosynthesis genes (glycoalkaloid metabolism, *GAMEs*) [63,64].

Anthocyanin-regulatory modules in fruit crops highlight tissue-specific JAZ-DELLA functions. MdJAZ2 and MdRGL2a dynamically regulate the MdNAC72-MdABI5 module to modulate anthocyanin production, according to recent studies in apples [65]; MdZFP7 acts as a new hub, integrating signals from the MdJAZ2-MdRGL3a interaction to regulate anthocyanin biosynthesis, while being degraded by the E3 ubiquitin ligase MdBRG3 [66]. Additionally, they work together to suppress the WD-repeat/bHLH/MYB complex, which inhibits the production of anthocyanins and the commencement of trichomes triggered by JA [4]. Research has demonstrated that JAZ and DELLA repressors target some bHLH components and R2R3 MYB components [67,68]. Furthermore, JAZ proteins can interact with MYC2/3/4/5 to control stamen development, and DELLA and JAZ work together to restrict the transcriptional stimulation of bHLH transcription components MYB21/24. This reduces their interactions with MYC2/3/4/5 and further suppresses filament elongation [69,70,71]. When GA is not present, DELLA can restrict the production of JA by preventing the expression of *DAD1* and *LOX* [60,72]. In response to cold stress in grapes, the GRAS transcription factor VaPAT1, which is antagonistically controlled by JA and GA, combines with IDD3 to upregulate *LOX3*, improving JA production and freezing tolerance [73].

Hormonal network rewiring exhibits notable dynamics during soybean flowering under field conditions. JA and GA engage in a temporally segregated antagonistic interaction throughout floral development: GA primarily drives early floral transition, whereas JA becomes activated during peak anthesis and may suppress GA-mediated pathways, ultimately balancing developmental progression with stress resilience [74].

### 2.4. JA and Ethylene Crosstalk: JAZ, MYC2, and EIN3

Ethylene is a plant hormone that affects how plants grow and develop, namely seed germination, fruit maturation, aging abscission, cell elongation, and resistance to necrotrophic fungal diseases [75]. The classical ethylene signaling pathway includes a group of ethylene receptors (ETR1/ETR2/EIN4/ERS1/ERS2), the protein kinase Constitutive Triple Response 1 (CTR1), the transmembrane protein Ethylene-Insensitive 2 (EIN2), and transcription elements Ethylene-Insensitive 3 (EIN3), EIN3-Like (EIL1/2), and Ethylene Response Factors [76]. When ethylene is present, receptor inactivation reduces EIN2 activation by CTR1, inhibiting ubiquitination and destruction via the proteasome pathway. This process enhances the accumulation of EIN3/EILs, which stimulates ERF transcriptional activity and regulates subsequent genes [77].

The JAZ proteins, MYC2, and the EIN3 transcription factor are the major nodes of the JA–ethylene signaling interaction (Figure 1D). The breakdown of JAZ proteins releases EIN3/EIL1, which are inhibited by the histone deacetylase RPD3 (HDA6), activating the expression of other ERF transcription factors (e.g., ERF1/ORA59) that regulate root hair development and bacteria response, such as *PDF1.2* [78,79]. MYC2 suppresses EIN3-induced HLS1, which regulates hook development, while also activating EIN3 Binding F-BOX Protein 1 (EBF1), which promotes EIN3 degradation via SCFEBF1 [80]. In addition, the expression of the wound- and herbivory-responsive gene *VSP2*, which is activated by MYC2, is repressed [78]. Crosstalk frequently involves the dynamic control of the JA–ethylene module. Ethylene causes the transcription factors SlERF15/16 to upregulate *LOXD*, *AOC*, and *OPR3*, boosting JA production in tomato in response to herbivory [81]. In peach, MeJA activates *PpSAMS*, *PpACS3/4*, and *PpACO*, which promotes ester biosynthesis and improves cold tolerance [82]. In apples, JA can interact with MdMYC2 and MdERF2 to block their interaction with MdERF3, or it may bind directly to the promoters of MdACS1/MdACO1 to activate transcription factor activity, inducing MdACS1 expression and boosting ethylene levels [83].

Furthermore, specific transcription factors can regulate JA and ET synergistically or antagonistically, acting as crosstalk points in plant defense systems. The transcription factor MED25 stimulates MYC2 and EIN3/EIL1, which then activate downstream pathways [84]. The *Arabidopsis* CCCH protein C3H14 activates ORA59 in response to pathogen defense via a WRKY33-dependent mechanism. WRKY33 also forms a transcriptional complex with ERF1, using the JA–ethylene route to upregulate the expression of phytoalexin biosynthesis genes *CYP71A13* and *PAD3*, therefore triggering phytoalexin synthesis in response to pathogen infections such as Botrytis cinerea [85,86,87]. Defense against necrotrophic infections is negatively regulated by WRKY57 and WRKY33’s competition for interaction with JAZ5/7. The BIG protein activates ERF1/ORA59 to positively control resistance against infections like Botrytis cinerea and suppresses MYC2 activity to negatively regulate herbivory resistance [88]. Herbivory defense mechanisms are adversely regulated by the Polycomb Repressive Complex (PRC1) protein LHP1, which mainly suppresses the activity of the MYC2 branch ANAC019/055 and *VSP1/2* genes [89].

### 2.5. JA and SA Crosstalk: MYC2, NPR1

SA has an important function in plant growth, development, and immunity. The primary SA receptors are NPR1 and its paralogs (NPR3/4), which orchestrate SA signaling through dynamic interactions with TGA transcription factors to activate pathogenesis-related (PR) genes. This process involves two coordinated mechanisms: SA perception by NPR1 triggers its translocation to the nucleus, where it forms an active complex with TGAs to directly induce PR gene expression; concurrently, NPR3/4 sense elevated SA levels and mediate NPR1 degradation via the CUL3-NPR3/4 E3 ubiquitin ligase complex, thereby modulating the signal amplitude and duration. This dual regulatory axis ensures the precise control of SA-responsive defenses while preventing hyperactivation [90,91,92,93].

MYC2 and NPR1 are the major nodes of crosstalk between JA and SA (Figure 1E). Current research indicates that these two hormones largely act antagonistically to modulate resistance to biotrophic and necrotrophic diseases. Crucially, MYC2 displays the dual regulation of SA accumulation: when Pseudomonas syringae releases COR to infect plants, MYC2 dynamically regulates SA levels by boosting the expression of the *ICS1* gene, which accumulates SA. It can also activate NACs (ANAC19/55/72), transcription factors that inhibit SA synthesis genes (e.g., *ICS1*), while boosting the expression of BSMT1, which can adversely control SA accumulation and depress plant defense [94,95]. NPR1 orchestrates dynamic competition: it interacts with MYC2 to prevent it from binding to MED25, decreasing pathogen defense and upregulating SA genes to coordinate immune responses [96,97]. In the rice nucleus, the OsbHLH6 transcription factor competes with and activates the MYC2 pathway while binding to OsNPR1 to interfere with its interaction with OsTGA transcription factors, dynamically regulating JA-SA defense responses [98]. In contrast, the PRC1 protein LHP1 boosts SA accumulation while inhibiting MYC2-mediated herbivory defense to increase resistance to Pseudomonas syringae [89]. SA can also decrease the activity of the ORF59 transcription factor in an SCFCOI1-JAZ-independent manner, effectively reducing the ERF branch of the JA defensive response [99].

Additionally, JA and SA antagonistically regulate WRKY-mediated defense and senescence responses [100]. The protein kinase MPK4 is considered a positive regulator of the JA network and a negative regulator of the SA network. Moreover, the SA-induced glutaredoxin GRX480 interacts with TGAs to inhibit the expression of the JA pathway gene *PDF1.2* [101]. EDS1 and its partner PAD4 complex interact with MYC2, the main regulator of the SA-JA pathway, to inhibit its activity and stimulate *ICS1* gene expression, thereby regulating plant defense against pathogens [102]. When exogenous MeJA is given to *Arabidopsis*, the zinc finger protein OZF1 positively controls JA signaling and SA-JA crosstalk, but it upregulates PR expression in response to SA [103]. In *Arabidopsis*, β-aminobutyric acid (BABA)-induced resistance (BABA-IR) involves the PBF-mediated downregulation of MYC2, which may shift the balance toward SA-dominated immunity [104]. Conversely, we can suppose that ERF2 promotes JA biosynthesis by activating the LOX and AOS genes, enhancing necrotroph defense without compromising SA pathways in *Hordeum vulgare* [105]. The phosphorylation of the Gα subunit GPA1 stabilizes the TCP14-JAZ3 complex, thereby counteracting pathogen effector HopBB1-mediated degradation; this mechanism concertedly suppresses JA signaling while enhancing SA-mediated immunity [106].

### 2.6. JA and SL Crosstalk

SL is a type of plant hormone generated from carotenoids that regulates root growth, lateral branching, reproductive development, leaf senescence, and defense mechanisms [107]. The signaling mechanism of strigolactones (SLs) is as follows: the receptor D14 (an α/β-fold hydrolase) and its homologs detect SLs, causing D14 to alter conformation. This alteration allows D14 to interact with the F-box protein MAX2/D3, resulting in the formation of a D14-SCFD3/MAX2 protein complex. The complex then binds and ubiquitinates D53, eventually creating the D53-D14-SCFD3/MAX2 complex. This process promotes the ubiquitin-mediated degradation of the transcriptional repressor D53/SMXLs (belonging to the SMXL protein family) through the 26S proteasome path, which activates downstream gene transcription [108,109,110].

**Figure 2:**
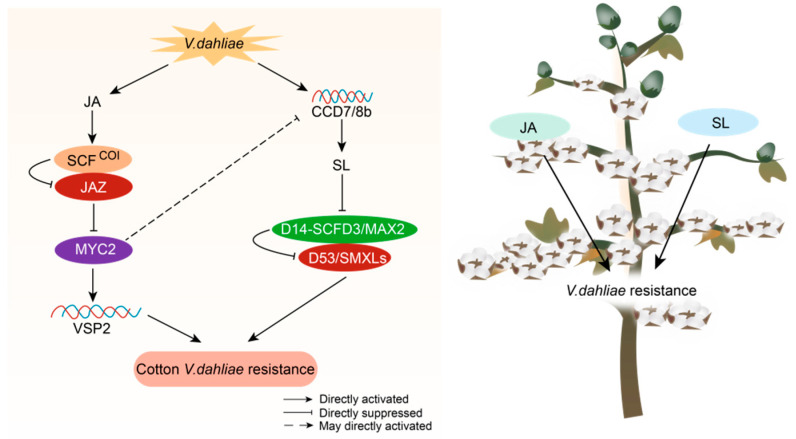
Molecular mechanisms of jasmonic acid and SL crosstalk in cotton. When cotton is infected with Verticillium wilt, JA and SLs act in concert to mediate defense responses. This is primarily achieved by modulating the levels of JA and SLs, which in turn affect the signaling pathways. The process involves a negative feedback loop of SL-GbMYC2-GbCCD7/8b and the histone deacetylase GhHDA5, both of which regulate defense responses.

In *Arabidopsis thaliana*, the crosstalk between JA and SLs has received less attention than other hormone interactions. Cotton research in recent years has found a JA-SL crosstalk network, comprising a negative feedback loop of SL-GbMYC2-*GbCCD7/8b* and a negative regulatory node involving the histone deacetylase GhHDA5, which regulates plant resistance to Verticillium wilt (Figure 2). When Gossypium barbadense is infected with Verticillium dahliae, GbMYC2 expression increases. GbMYC2 binds to the promoters of *GbCCD7* and *GbCCD8b*, which encode the rate-limiting enzymes for SL biosynthesis, blocking their expression and dynamically controlling the SL balance in root cells [111]. In *Gossypium hirsutum*, the overexpression of *GhHDA5* results in elevated JA levels and decreased SL levels, negatively affecting resistance to Verticillium wilt; the underlying molecular mechanisms remain to be elucidated, but it may occur in petals, pistils, and stamens [112]. In rice, SLs prevent the formation of JA and JA-Ile after mechanical injury, whereas, in melon, SLs enhance JA accumulation via increasing *LOX* gene expression in response to cadmium stress [113]. Studies in tobacco have demonstrated that SL signaling repressors (e.g., NaSMXL6/7) directly interact with NaJAZ proteins, accelerating their degradation and releasing downstream signals such as NaMYC2 for expression [114,115,116]. The exogenous application of JA substantially inhibits SL accumulation in longan by upregulating DlSMXL6 [117].

The observed divergence in JA-SL interactions across species likely reflects evolutionary adaptations to distinct ecological pressures. In cotton, the SL-GbMYC2-GbCCD7/8b negative feedback loop may optimize resource allocation during Verticillium defense: suppressing SL biosynthesis under pathogen attack (via GbMYC2) prioritizes JA-mediated immunity while conserving resources for root development—a critical trait in perennial crops facing soil-borne diseases [111]. Conversely, in monocots like rice, the SL-mediated suppression of JA after mechanical wounding (e.g., herbivory) could balance growth–defense trade-offs in resource-limited environments [113].

### 2.7. JA and BR Crosstalk

Brassinosteroids (BRs) are essential for plant growth and development, as well as responding to abiotic stressors. In the absence of BR signaling, the BR signaling inhibitor BRI1 kinase inhibitor 1 (BKI1) inhibits the activity of the plasma membrane receptor kinase brassinosteroid insensitive 1 (BRI1). When BRs are present, BRI1 becomes stimulated and interacts with its co-receptor, BRI1-associated receptor kinase 1 (BAK1), to form a complex that phosphorylates itself. This process causes BKI1 to be released, which activates the signaling kinases BR-signaling kinase 1 (BSK1) and BRI1 suppressor 1 (BSU1), inhibiting the negative regulator BIN2 [118,119]. This cascade activates BES1/BZR1, which translocate to the nucleus and bind to the promoters of downstream target genes, regulating their expression [118,119].

**Figure 3:**
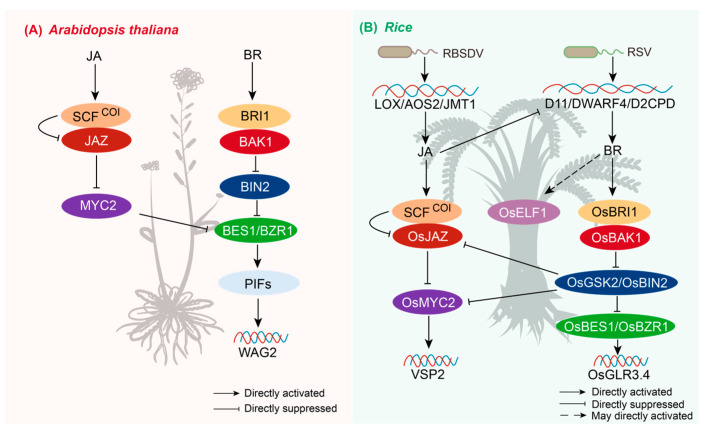
Molecular mechanisms of jasmonic acid and BR crosstalk. (**A**) In *Arabidopsis thaliana*, JA and BR antagonistically regulate apical hook absence and root development. WAG2: a gene encoding apical hook absence and cotyledon opening. (**B**) In rice, crosstalk mainly responds to viral defense processes. OsGLR3.4: encodes a glutamate receptor-like protein, a direct target gene of OsBZR1, regulating growth and defense; LOX1, AOS2, JMT1: genes involved in JA biosynthesis; D11, OsDWF4, D2, CPDs: genes involved in BR biosynthesis; PR1b/PR5: defense-related genes responding to rice blight.

The antagonistic interaction between JA and BRs is primarily manifested in the dynamic co-regulation of signaling transduction, which controls plant growth, development, and defense responses to environmental or biotic stresses (Figure 3). During developmental processes, these hormones typically display antagonism. For instance, in *Arabidopsis*’s hypocotyl development, the JA-activated transcription factor MYC2 binds to BZR1, a key regulator of BR signaling. This binding disrupts the BZR1-PIF4 complex, consequently suppressing the expression of the downstream gene *WAG2*. This mechanism inhibits apical hook formation and promotes cotyledon opening. Conversely, BR counteracts this JA effect by stabilizing the BZR1-PIF4 interaction (Figure 3A) [120]. In the context of root growth regulation, BR suppresses JA-mediated root growth inhibition, while JA downregulates BR biosynthesis genes, such as *DWARF4 (DWF4)* and *CPD*, via COI1-dependent signaling [121].

However, JA and BR often act synergistically to enhance plants’ defense capabilities against biotic stresses. This cooperative relationship is particularly evident in rice responses to viral infections. Researchers demonstrated that the gene *OsGLR3.4*, directly targeted by BZR1, is an important link in rice growth, regulated by BR and involved in the JA defense network, although the specific molecular mechanisms remain unclear (Figure 3B) [122]. Recently, studies in rice have revealed that, during infection by Rice Stripe Virus (RSV), BR inactivates OsGSK2, a negative regulator in its signaling pathway. This inactivation releases the transcription factor OsMYC2, which is normally suppressed by OsGSK2, thereby activating JA-dependent defenses. Conversely, during infection by Rice Black-Streaked Dwarf Virus (RBSDV), OsGSK2 promotes the degradation of JA signaling repressors OsJAZ4 and OsJAZ11, thereby enhancing JA responses. Notably, in the RBSDV context, JA simultaneously antagonizes the BR pathway by suppressing BR biosynthesis genes (*D11/OsDWF4/D2CPD*) while upregulating JA biosynthesis genes (*LOX1/AOS2/JMT1*), highlighting the regulatory complexity [123,124,125]. In bacterial blight resistance, the BR pathway key transcription factor OsELF1 and the JA pathway antagonistically regulate the transcription factor OsWRKY4. Nevertheless, both pathways converge to induce the expression of pathogenesis-related genes *PR1b* and *PR5* in response to rice blight [126].

The plasticity of this JA-BR interplay is further demonstrated in adaptive reprogramming to environmental changes. During pear bud dormancy release, low-temperature stress triggers synergy through the formation of PpyBZR2-PpyMYC2 heterodimers, which co-activate genes involved in dormancy breaking [127]. Core regulators GSK2/BIN2 and MYC2 exhibit species- and stress-dependent functional reversibility, underpinning the plasticity of JA-BR crosstalk, which dynamically balances resource allocation between growth and defense.

### 2.8. JA in Multi-Stress and Symbiotic Contexts

JA signaling exhibits multi-layered coordination in symbiotic interactions under abiotic stress. Transcriptomic analyses of Septoglomus constrictum-inoculated tomato reveal the concerted upregulation of JA biosynthesis/signaling genes (e.g., LOX, JAZ) with ethylene and auxin pathways in both leaves and roots under heat stress. This demonstrates that mycorrhizal symbiosis enhances plant adaptation to compound stress through integrated phytohormonal networks (JA–ethylene–auxin), extending the molecular framework of JA in systemic stress coordination [128].

### 2.9. JA-Mediated Chromatin Remodeling

The epigenetic regulation of jasmonate (JA) signaling hinges on dynamic chromatin remodeling, with two central mechanisms identified. The first is JAZ-PcG complex-mediated repressive modification: JAZ proteins interact with Polycomb group (PcG) complexes (e.g., EMF2, LHP1) to recruit H3K27me3 methyltransferase CLF, establishing repressive chromatin states at loci critical for stamen development (e.g., DYT1, AMS) and JA-responsive genes. JA-induced JAZ degradation via COI1 releases PcG suppression, activating downstream transcriptional networks to balance development and defense [129]. The second is MED25-MYC2-dependent chromatin looping: JA orchestrates enhancer–promoter interactions dynamically through the mediator subunit MED25. For instance, the enhancer ME2 at the MYC2 locus forms chromatin loops to activate MYC2 during short-term JA responses but switches to repression under sustained JA, forming a feedback circuit [130,131].

While the roles of JA in epigenetic pathways are emerging, networks governing synergistic or antagonistic multi-hormone interactions are largely unknown. Key insights include the following: the mediator complex integrates JA signaling with other hormones via subunits (e.g., MED25, MED16). MED16 stabilizes MED25 by inhibiting its degradation by E3 ligases MBR1/2, sustaining JA signaling [132]. MED25 interacts with JAZ proteins and MYC2, providing a paradigm for dynamic hormonal control. JA promotes defense gene activation via MED25-HAC1-mediated H3K9 acetylation. Antagonistically, repressive H3K27me3 modification may suppress this activation in auxin or ABA signaling pathways [133].

## 3. Conclusions and Future Perspectives

Plant growth and development require coordinated hormone signaling, with the jasmonate (JA) pathway critically regulating environmental responses and growth–defense balance. JA interacts with abscisic acid (ABA), auxin, gibberellin (GA), ethylene (ET), brassinosteroids (BRs), strigolactones (SLs), and salicylic acid (SA) to form a complex adaptive network. Each type of JA–hormone crosstalk involves specific molecular nodes. JA-ABA is centered on transcription factor MYC2 (integrating signals, activating defense/drought genes) and JAZ repressors (modulating MYC2 and ABI3/4/5 activity) [33,34]. In JA–auxin, key nodes are JAZ proteins and the MYC2/ARF transcription factors, regulating processes like root growth and senescence. JA-GA is mediated by JAZ-DELLA protein interactions, impacting growth, development, and anthocyanin production. In JA-ET, core nodes are transcription factors MYC2 and EIN3/EIL1, dynamically controlling development and stress tolerance. JA-SA is governed by MYC2 and NPR1, regulating the activation and suppression of defense pathways against pathogens. JA-BR and JA-SL primarily involve interactions between JAZ proteins and hormone-responsive transcription factors (e.g., GSK2/BIN2 for BR, SMXL for SL), although specific contact points are less well defined.

While our understanding of the molecular mechanisms underlying JA crosstalk with other hormones has advanced, many key links remain unclear. Crosstalk operates through three principal layers: (i) the reciprocal modulation of biosynthetic genes—JA represses *NCED* to lower ABA and break seed dormancy [21]; (ii) TF–protein interactions—JAZ-DELLA complexes attenuate filament elongation by inhibiting MYB21/24 transcription [71]; (iii) direct gene regulation—the blockade of ERF1/ORA59 sharply reduces *PDF1.2* expression and compromises disease resistance [79].

Research on epigenetic regulation in hormone crosstalk remains sparse, despite existing studies indicating its primary concentration at the transcriptional level. Epigenetic modifications—including DNA methylation, histone modifications, and mediator complex regulation—offer adaptable, reversible mechanisms for long-term gene expression control.

Elucidating the molecular mechanisms of the crosstalk between jasmonates and other plant hormones at the transcriptional regulation level is crucial in understanding plant growth and development (e.g., seed germination, flowering, fruit maturation), as well as stress responses (e.g., resistance to insects, pathogens, and drought). Building on our synthesis of core molecular mechanisms, future efforts should prioritize mapping spatiotemporal dynamics (e.g., single-cell hormone imaging) and epigenetic layers (e.g., CRISPR-based editing of histone marks) to resolve context-specific crosstalk. To achieve the deeper elucidation of the precise spatial distribution, in situ sites of action, and subsequent systemic cascades of jasmonate (JA) and other phytohormones, rapidly evolving spatial molecular biology and in situ technologies—such as single-cell RNA sequencing (scRNA-seq), spatial transcriptomics, and high-resolution in situ imaging [134]—will provide indispensable and transformative perspectives. Validating these interactions in crops (e.g., via JAZ gene editing in millet) will bridge mechanistic knowledge with agricultural applications.

## Data Availability

All data used in this review paper are available online.
